# Evaluation of developmental changes in bovine in vitro produced embryos following exposure to bovine *Herpesvirus* type 5

**DOI:** 10.1186/1477-7827-10-53

**Published:** 2012-07-23

**Authors:** Mariana PC Brenner, Camila Silva-Frade, Marina C Ferrarezi, Andrea F Garcia, Eduardo F Flores, Tereza C Cardoso

**Affiliations:** 1 UNESP, Laboratory of Animal Virology, University of São Paulo State, College of Veterinary Medicine, Araçatuba, SP, 16050-680, Brazil; 2 Departament of Preventive Veterinary Medicine, Federal University of Santa Maria, UFSM, College of Veterinary Medicine, Santa Maria, RS, 97115-900, Brazil

**Keywords:** BoHV-5, Infection, Bovine embryos, Apoptosis

## Abstract

**Background:**

Bovine *Herpesvirus* type-5 (BoHV-5) is a neurovirulent α-*Herpesvirus* which is potentially pathogenic for cows and suspected to be associated with reproductive disorders. Interestingly, natural transmission of BoHV-5 by contaminated semen was recently described in Australia. Additionally, BoHV-5 was also isolated from the semen of a healthy bull in the same country and incriminated in a natural outbreak of reproductive disease after artificial insemination. In contrast with BoHV-1, experimental exposure of in vitro produced bovine embryos to BoHV-5 does not affect embryo viability and seems to inhibit some pathways of apoptosis. However, the mechanisms responsible for these phenomena are poorly understood. In this study, we examined mitochondrial activity, antioxidant protection, stress response and developmental rates of in vitro produced bovine embryos that were exposed and unexposed to BoHV-5.

**Methods:**

For this purpose, bovine embryos produced in vitro were assayed for cell markers after experimental infection of oocytes (n = 30; five repetitions), in vitro fertilization and development. The indirect immunofluorescence was employed to measure the expression of superoxide dismutase 1 (SOD1), anti-oxidant like protein 1 (AOP-1), heat shock protein 70.1 (Hsp 70.1) and also viral antigens in embryos derived from BoHV-5 exposed and unexposed oocytes. The determination of gene transcripts of mitochondrial activity (*SOD1*), antioxidant protection (*AOP-1*) and stress response (*Hsp70.1*) were evaluated using the reverse transcriptase polymerase chain reaction (RT-PCR). MitoTracker Green FM, JC-1 and Hoechst 33342-staining were used to evaluate mitochondrial distribution, segregation patterns and embryos morphology. The intensity of labeling was graded semi-quantitatively and embryos considered intensively marked were used for statistical analysis.

**Results:**

The quality of the produced embryos was not affected by exposure to BoHV-5. Of the 357 collected oocytes, 313 (+/− 6.5; 87,7%) were cleaved and 195 (+/− 3.2; 54,6%) blastocysts were produced without virus exposure. After exposure, 388 oocytes were cleaved into 328 (+/− 8.9, 84,5%), and these embryos produced 193 (+/− 3.2, 49,7%) blastocysts. Viral DNA corresponding to the US9 gene was only detected in embryos at day 7 after in vitro culture, and confirmed by indirect immunofluorescence assay (IFA). These results revealed significant differences (*p* < 0.05) between exposed and unexposed oocytes fertilized, as MitoTracker Green FM staining Fluorescence intensity of Jc-1 staining was significantly higher (*p* < 0.005) among exposed embryos (143 +/− 8.2). There was no significant difference between the ratios of Hoechst 33342-stained nuclei and total cells in good-quality blastocysts (in both the exposed and unexposed groups). Using IFA and reverse transcriptase polymerase chain reaction (RT-PCR) for the set of target transcripts (*SOD1, AOP-1 and Hsp 70.1*), there were differences in the mRNA and respective proteins between the control and exposed embryos. Only the exposed embryos produced anti-oxidant protein-like 1 (*AOP-1*). However, neither the control nor the exposed embryos produced the heat shock protein *Hsp 70.1*. Interestingly, both the control and the exposed embryos produced superoxide dismutase (*SOD1*), revealing intense mitochondrial activity.

**Conclusion:**

This is the first demonstration of *SOD1* and *AOP-1* production in bovine embryos exposed to BoHV-5. Intense mitochondrial activity was also observed during infection, and this occurred without interfering with the quality or number of produced embryos. These findings further our understanding on the ability of α-*Herpesviruses* to prevent apoptosis by modulating mitochondrial pathways.

## Background

Many calves are produced by in vitro embryo production (IVP) and transferred worldwide [1]. However, the production of blastocysts from mature in vitro oocytes has only a modest success rate [2]. Despite many efforts to improve IVP techniques, the successful development of only 30% to 40% of blastocysts appears to be the limit [1]. A number of factors can influence the culture environment, such as media composition, protein supplementation, the number of embryos present in the culture drop and gas atmosphere. Among those factors, oxidative stress induced by greater oxygen tension has received special attention in the last few years [3-6]. Generally, higher oxygen (O_2_) tension (5% CO_2_ in air) than that of the *in vivo* environment employed for in vitro culture (IVC) is responsible for the increased generation of reactive oxygen species in cells, which can lead to DNA damage, lipid peroxidation and oxidative modification of proteins [4]. The role of mitochondria in the all-aerobic cell system has been widely studied, and these organelles play a well-known role as the energy-ATP resource for most of the reactions occurring in cells, including metabolic pathways, fatty acid metabolism, urea metabolism and the metabolism of specific hormones [7].

Concerning to oocytes and embryos, mitochondria are important organelle not only for competence but also for adequate reproduction [8]. During early development, the role of maternal mitochondria dominates over paternal mitochondria because sperm mitochondria are ubiquitinated and eliminated when the sperm enters the ooplasm [9]. Although distinct patterns of mitochondrial distribution and activity have been described at different stages of bovine embryonic development, it is still an important parameter for assessing the potential competence of the oocytes and embryos [9].

Latent infection is a hallmark of the *Herpesviridae* family [10]. It is noteworthy that BoHV-5 can be reactivated from a persistent state and excreted without clinical signs, consistent with the low incidence of apoptotic embryos derived from oocytes exposed to virus infection in previous studies [11,12]. Moreover, cattle are considered the natural host of BoHV-5, and latently infected animals constitute natural reservoirs of the virus [13-15]. Thus, biological products derived from latently infected sources may be potentially contaminated with the virus and, therefore, represent a potential source of contamination for IVC procedures [12]. Despite numerous reports of BoHV-5 in bull semen, the significance of BoHV-5 in semen is unknown [16-20]. Recently, an association between venereal disease in cows and artificial insemination with semen contaminated with BoHV-5 has been described in Australia [18,21].

Viral infection typically results in the perturbation of cellular processes that can serve to trigger cell death via the mitochondrial pathway [18]. Successful replication of many viruses, therefore, depends on the ability of the virus to prevent apoptosis induced by the mitochondrial pathway [7]. The maintenance of mitochondrial respiration during viral infection is essential for ensuring that sufficient ATP is available for viral replication to proceed, while concomitantly inhibiting apoptosis induced by oxidative stress [22]. Interestingly, experimental infection of bovine gametes with BoHV-5 led to the infection of in vitro-produced embryos without interference to embryonic development, and infection with BoHV-5 suppressed specific apoptotic pathways [11,23]. Determining how BoHV-5 interferes with cell-death pathways will not only improve our understanding of viral pathogenesis but also has the potential to advance our understanding of the processes that normally control of cellular death pathways. For example, oxidative stress is essential for apoptotic induction to proceed in response to many stimuli [23]; however, the mechanisms by which BoHV-5 inhibits bovine embryo apoptosis or induces mitochondrial dysfunction are unknown.

The objective of this study was to investigate the role of mitochondrial activity, antioxidant protection, and the stress response in bovine embryos exposed to BoHV-5 and healthy IVC embryo controls. For this purpose, bovine oocytes were experimentally exposed to BoHV-5. The presence of viral antigens, superoxide dismutase 1 (*SOD1)*, anti-oxidant like protein 1 (*AOP-1)* and heat shock protein 70.1 *(Hsp70.1)* in both exposed and unexposed embryos were confirmed by indirect immunofluorescence and by assessing transcription of related genes. BoHV-5 viable particles were recovered from infected embryos by virus re-isolation in MDBK cells and by polymerase chain reaction (PCR) targeting the US9 gene.

## Methods

### Reagents and media

Unless otherwise stated, all chemicals were obtained from Sigma-Aldrich (St. Louis, MO, USA) and Invitrogen (Grand island, NY, USA) at the highest available purity. The basic dilutions and culture media were cell culture grade, which are routinely used in our laboratory.

### Oocyte collection, in vitro fertilization and maturation

Oocyte collection, in vitro maturation (IVM), sperm and in vitro fertilization procedures were followed as previously described [11,12]. The study was conducted with presumptive zygotes produced from oocytes exposed or unexposed (n = 30/per slide; in five repetitions) to BoHV-5 (the latter underwent the same protocol but without exposure to BoHV-5). Frozen-thawed sperm for IVF were derived from 0.5 mL straws of bovine (*Bos indicus*) semen collected from a single bull. Semen was centrifuged on a Percoll (Nutricell®, Campinas, SP, Brazil) gradient at 700 x g for 20 min. The resulting sperm pellet was washed in TALP medium (Tyrode medium with added bicarbonate buffer and supplemented with 6 mg BSA per milliliter) and centrifuged at 200 X *g* for 5 min. The pellet was diluted in IVF medium (TALP medium supplemented with 3 mg/mL heparin and PHE solution: 2 mM penicillamine, 1 mM hypotaurine, and 250 mM epinephrine) to a final concentration of 1 x 10^5^ sperm/mL in drops of 100 μL. After 24 h of maturation, the oocytes were transferred to drops containing IVF medium. For IVF, oocytes and sperm were co-incubated in the IVF medium for 20 h under the same conditions used for IVM. Afterward, presumptive zygotes (PZ) were placed in in vitro culture medium (IVC) up to day 7 (day 0 = day of fertilization). After blastocyst production on day 7 post-fertilization, only embryos graded as Code 1 (Excellent or Good) or Code 2 (Fair) following the IETS guidelines [1] were used. Similarly, only oocytes and presumptive zygotes classified as good quality were used. All uninfected cells and reagents used in this study were assayed for bovine *herpesvirus* types 1 and 5 (BoHV-1 and 5), bovine viral diarrhea (BVD), and others pathogens, e.g., Mycoplasma by the use of molecular search [11].

### Virus infection and embryo development

Stocks of BoHV-5, isolated in 2007 from outbreaks in Araçatuba, SP, Brazil [24], were propagated in Madin-Darby bovine kidney (MDBK, ATCC CCL-2) cells, which were cultured in minimum essential medium (MEM) [25,26]. The tissue culture infective dose per 50 μL (TCID_50_) of stock virus was determined by virus titration infection of confluent monolayers of MDBK cells [25,26] at a multiplicity of infection (MOI) of 1. Aliquots of stock virus (100 μL) with 10^3.3^ TCID_50_/50 μL were frozen at −86°C prior to use. Only COCs with several layers of compact cumulus cells and homogeneous cytoplasm were used, divided into drops of 30 oocytes each for experimental use. The culture consisted of oocytes maintained in 100 μL TCM-199 (GIBCO-BRL, Grand Island, NY, USA), supplemented with 10% FBS, 2.2 mg/mL sodium bicarbonate, 0.02 mg/mL sodium pyruvate, 0.05 mg/mL gentamicin sulfate, 0.5 μg/mL FSH (Pluset, Calier, Barcelona, Spain), and 50 μg/mL LH (Lutropin-V, Bioniche Inc., Belleville, ON, Canada) for 24 h at 39°C in 5% CO_2_-air. Selected oocytes (n = 30) were washed in maturation medium consisted of TCM-199 (GIBCO-BRL, Grand Island, NY, USA) supplemented with 10% FBS (Nutricell®), 2.2 mg/mL sodium bicarbonate, 0.02 mg/mL sodium pyruvate, 0.05 mg/mL gentamicin sulfate, 0.5 μg/mL FSH (Pluset®, Calier, Barcelona, Spain) and 50 μg/mL LH (Lutropin-V®, Bioniche Inc., Belleville, ON, Canada). Oocytes were transferred to drops containing 100 μL of MM in five repetitions in a total number of 150 oocytes. Oocytes were experimentally exposed by co-incubation with 10 μL BoHV-5 (10^2.3^ TCID_50_ corresponding to 1 MOI) for 1 h at 39°C in 5% CO_2_-air. The oocytes were subsequently washed three times and transferred to new, virus-free maturation drops for further in vitro development, as previously described [11,12].

### BoHV-5 molecular detection

The viral DNA detection was applied to detect the *US9* gene among exposed and unexposed derived-embryos, follicular liquid, oocytes and sperm, as described previously [27]. A 250-bp amplicon was generated based on the sequence of US9 region (GenBank accession number AY064172). Procedures for DNA purification and PCR were previously described [10]. The PCR was performed using 12.5 μL of 2× *Platinum* Taq Polymerase High-Fidelity Master Mix (Invitrogen™^TM^, Carlsbad, CA, USA), and 10 pmoles of each primer (US9-For: 5’-AGAGTCCACACAGCGTCGTCAA-3’ and US9-Rev 5’-CTACAGCGAGAGCGACAGCGAGA-3’). The reverse primer (US9-Rev) was purchased by Invitrogen. In addition, 2.5 μL of nuclease-free water and 8 μL of the DNA sample (positive reference strain AY064172) consisting of 3 μL of DMSO plus 5 μL of DNA were added to the master mix. In an automated thermocycler (Eppendorf, Hamburg, Germany), the reactions were incubated at 98°C for 5 min; 34 cycles of 94°C for 30 s, 60°C for 1 min, and 72°C for 2 min, and finally 72°C for 5 min. The PCR products were visualized on a 1.5% (w/v) agarose gel after staining with SYBR green at 0.5 μg/mL of concentration.

### Virus isolation

Embryos derived from oocytes directly exposed to BoHV-5 and observed during in vitro production were collected and freeze-thawed (1 h under −86°C and 1 h under room temperature) three times. Monolayer cultures of MDBK cells at 80% confluence were prepared, according to standard procedures, to be free of BoHV-1 and any other potential pathogens [12]. Adsorption was allowed for 90 min at 38.5°C. Then, fresh medium was added, and for the next 7 dayays, cultures were examined for cytopathic effect (CPE). After an additional passage, the cultures with no evidence of CPE were considered negative. When CPE was observed, the respective cells were removed and submitted to virus titration and identification. Virus titration was conducted with infected embryo suspensions and 96-well plates previously seeded with MDBK cells. Serial dilutions, from 10^-2^ to 10^-8^, of infected embryos suspensions were prepared and used for BoHV-5 titration onto a single well in triplicate. The plate was incubated for 1 h at 38.5°C prior to adding 100 μL of supplemented MEM. The plates were incubated for 7 d and examined every 24 h for evidence of CPE. Infectious virus was calculated according to the Spearmann-Kärber method, as described [26].

### MitoTracker green FM, jc-1 and Hoechst 33342 staining

To evaluate the number of embryos/slide, intensively labeled with MitoTracker Green FM, Jc-1 and Hoechst 33342 probes, 30 selected embryos, in five repetitions, from oocytes exposed or unexposed to BoHV-5 were washed in PBS and then fixed with 4% (w/v) formaldehyde. This procedure was similar for all dyes used to assay exposed and unexposed embryos. MitoTracker Green FM (Invitrogen, Eugene, OR, USA) was used to evaluate mitochondrial distribution and segregation patterns. The MitoTracker Green FM was diluted in DMSO at 10 nM per slide direct applied on fixed embryos (30 per slide, in a total of 150 analyzed) and incubated for 10 min at 38.5°C. The mitochondrial distribution in the cytoplasm of embryos appeared as increased areas of fluorescence intensity or aggregates detected by fluorescence. Mitochondrial activity was qualified based on Jc-1 (5,5´, 6,6´-tetrachloro-1,1´, 3,3´-tetraethyl-benzimidazoyl-carbocyanine iodide) staining. Jc-1 monomers were detected with a green filter. Jc-1 dimers that formed on mitochondrial membranes with high potential were detected via a red filter. Mitochondria distribution in the embryonic cytoplasm was evaluated by the intensity of the green/red fluorescence. To observe the embryo quality, 1 μg/mL per slide of Hoechst 33342 at 38.5°C for 30 min was used. The fixed embryos were washed again to remove excessive Hoechst 33342 and then mounted onto slides under coverslips to evaluate the nuclear configuration. To measure the fluorescence intensity (MitoTracker Green FM emission 500 nm, Jc-1 red filter 515 nm and green filter 488 nm, and Hoechst 33342, emission 488 nm, stained slides were observed under an AxioImager A.1 light and ultraviolet microscope connected to an AxioCam MRc camera (Carl Zeiss, Oberkochen, Germany), and micrographs were processed with AxioVision 4.8 software (Carl Zeiss).

### Indirect immunofluorescence to assay *SOD1, AOP-1, Hsp70.1* and BoHV-5 antigens

Exposed and unexposed embryos (n = 30/slide; in five repetitions) were washed three times in PBS and fixed in 4% formaldehyde for 24 h at 4°C. The samples were then rinsed with PBS and permeabilized with proteinase *K* (10 μg/mL, Invitrogen) for 15 min at room temperature. After pre-treatment with proteinase *K* (10 μg/mL) at 4°C, the slides were incubated overnight with primary antibodies against mitochondrial superoxide dismutase, anti-oxidative protein 1 and stress response heat shock protein 7 (mouse anti-*SOD1*; anti-*AOP-1* and anti-*Hsp70.1*, respectively) diluted 1:50 in antibody diluent (PBS plus 0.1% of Tween 20). The viral antigens were detected by reacting exposed and unexposed embryos to monoclonal anti-BoHV-5 diluted in PBS plus 0.1% Tween 80 at 1:5 [26]. The slides were then incubated for 24 h at 4°C with secondary antibody (FITC-goat anti-mouse IgG; Zymed, South San Francisco, CA, USA). Omission of the primary antibody was used as a negative control. Subsequently, all samples were counterstained with 1 mg/mL of DAPI (4`-6-diamino-2-phenylindole; Sigma-Aldrich®) for 15 min at room temperature before mounting the slides in the dark [11].

### Determination of gene transcripts of mitochondrial activity (*SOD1*), antioxidant protection (*AOP-1*) and stress response (*Hsp70.1*)

The abundance of transcripts for genes related to mitochondrial activity antioxidant protection, manganese-superoxide dismutase (*SOD1*) sense 5´-CCCATGAAGCCTTTCTAATCCTG-3´ and antisense 5´-TTCAGAGGCGCTACTATTTCCTTC-3´ primers (accession no. L22092.1) and antioxidant protein like 1 (*AOP-1*) sense 5´-CCTAGGTTATTTAGCGCGT-3´ and antisense 5´-TTTCCGCTAGCGCTTATT-3´primers were evaluated using the reverse transcriptase polymerase chain reaction (RT-PCR), and these primers generated amplicons of 297 and 310 bp, respectively. The stress response gene transcription was performed by amplification of heat shock protein 70.1 (*HSP 70.1* accession no. U09861) sense 5´-AAGGTGCTGGAGTAGGCT-3´ and antisense 5´-ACTTGGAAGTAAACAGAAGC-3´primers producing an amplicon of 312 bp. Total RNA was isolated from 7 pools of 10 exposed and unexposed embryos using the PureLink® viral RNA/DNA extraction kit, according to the manufacturer’s protocol (Invitrogen). The total RNA was eluted in 20 μL of ultra-pure water and treated with 0.5 IU DNAse. The reverse transcriptase reaction (RT) was immediately performed using 0.5 μg oligo (dT) primers (Invitrogen). The reaction mix consisted of 200 μM of each dNTP, 1 x RT buffer, 2 μL DTT 0.1 M, 40 IU RNase inhibitor and 200 IU SuperScript II (Invitrogen). The RT reaction was performed at 42°C for 52 min, with a final incubation at 70°C for 15 min. Polymerase chain reaction was conducted, as previously described in the Materials and Methods section. The PCR products were visualized on a 1.5% (w/v) agarose gel after staining with SYBR green (Invitrogen) at 0.5 μg/mL of concentration.

### Semi quantitation and data analysis

The levels of *SOD1, AOP-1, Hsp70.1* and viral antigens were semi-quantitated according to the intensity of the immunofluorescence reactions. Two standard filters were employed: a DAPI filter (emission wavelength: 425 nm) was used to determine quality and a fluorescein isothiocyanate (FITC) filter (emission wavelength, 512 nm) was used to detect *SOD1, AOP-1, Hsp70.1* and BoHV-5 antigens. Bovine embryos were examined on two separate occasions by two observers without prior knowledge of the classification. The intensity of labeling was graded and only embryos considered marked lebeled were included in a semi-quantitative analysis. Differences in the respective mean values (n = 30 in five repetitions) were tested using ANOVA, with the primary effects as morphological quality groups, followed by a multiple pair-wise comparison using Student’s *t*-test for independent samples and the Bonferroni *t*-test. Differences of *P* < 0.05 were considered significant. The images were collected under an AxioImager A.1 light and ultraviolet microscope connected to an AxioCam MRc (Carl Zeiss, Oberkochen, Germany), and the micrographs were processed using the Axiovision 4.7 software (Carl Zeiss). The results are expressed as the mean +/− S.E.M. p values < 0.05 were considered significant.

## Results

### Embryo development and virus detection

**Figure 1:**
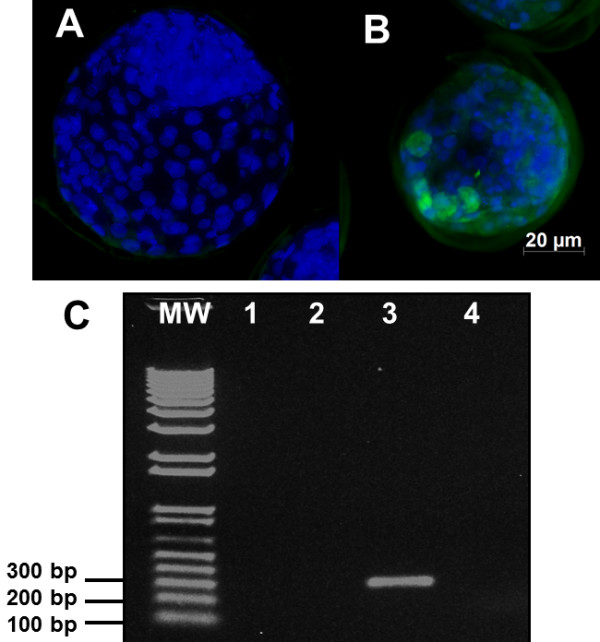
**Day 7 in vitro cultured bovine blastocysts derived from control oocytes (unexposed to BoHV-5) and exposed oocytes. A**) DAPI labeling (425 nm) under fluorescence shows the good quality of the exposed embryos; **B**) Indirect immunofluorescence assay showing positive signals to BoHV-5 antigens under fluorescence (512 nm). Scale bars is equal to100 μm; **C**) An electrophoresis agarose gel of US9 PCR: MW-molecular weight 1-kb plus; lanes 1 and 2 represent PCR results from unexposed oocytes and bovine semen; lane 3 shows the positive PCR amplification of 7-day embryos derived from exposed oocytes (250 bp); lane 4 shows the negative PCR amplification of embryos derived from unexposed oocytes.

From 357 unexposed oocytes and 388 oocytes exposed to BoHV-5 infection, 131 +/− 6.5 and 328 +/− 8.9 were cleaved, respectively (Table 1). There was no significant difference relative to the total number of unexposed oocytes when compared with those exposed to BoHV-5. There was no significant effect of virus infection on embryonic development, including proportions of oocytes that developed into blastocysts: unexposed 195 +/− 3.2 (54,6%) and exposed 193 +/− 3.2 (49,7%). The quality of the infected and uninfected embryos was considered to be similar, and both groups were rated as Code 1 (excellent or good). To confirm virus infection, BoHV-5 antigens were identified by indirect immunofluorescence assay (IFA) and PCR amplification in all infected embryos (Figure 1B and C, line 3). No evidence of BoHV-5 infection was observed among the unexposed oocytes (Figure 1C, line 1), sperm (Figure 1C, line 2) and presumptive embryos (Figure 1C, line 4). BoHV-5 was recovered from exposed embryos after infection of MDBK monolayers (data not shown).

**Table 1:** Comparison of development rates of exposed and unexposed bovine embryos submitted to BoHV-5 unexposed or exposure

**Groups**	**Total number of oocytes**	**Embryonic development (mean ± sd)**	
		**Cleaved, n (%)**	**Blastocysts, n (%)**
*Unexposed*	357	313 ± 6.5^a^ (87,7)	195 ± 3.2^a^ (54,6)
*Exposed*	388	328 ± 8.9^b^ (84,5)	193 ± 3.2^a^ (49,7)

^a,b^ Within a column, values without a common superscript differ (*p* < 0.05).

### Expression of MitoTracker green FM, jc-1 and Hoechst 33342 labels

**Figure 2:**
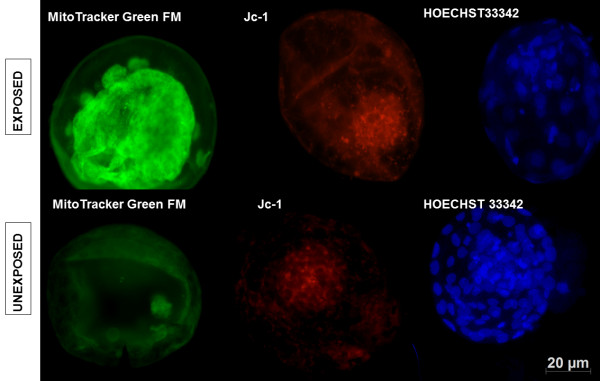
**Day 7 in vitro cultured bovine blastocysts derived from oocytes exposed and unexposed to BoHV-5.** MitoTracker Green FM labeling under fluorescence (500 nm) demonstrates an intense signal inner cell mass of exposed embryos; Jc-1 labeling under fluorescence (515–488 nm) reveals an intense signal in exposed embryos; Hoechst 33342 staining under fluorescence (488 nm) illustrates no difference between the qualities of exposed and unexposed embryos. Scale bar is equal to 20 μm.

The results revealed differences (*p* < 0.05) between exposed (123 +/− 8.5) and unexposed (53 +/− 3.9) for MitoTracker Green FM staining (Figure 2; Table 2). Cytoplasmatic fluorescence intensity was higher in exposed embryos (inner mass) than in unexposed ones (Figure 2). In addition, in the results observed for Jc-1 labeling exposed embryos had an intense red label, which was different (143 +/− 8.2; *p* < 0.05) from that of unexposed embryos (Table 2; Figure 2). Moreover, among the exposed and unexposed embryos, three patterns of mitochondrial distribution were observed: (1) Jc-1 staining was diffuse with mitochondria distributed throughout the cytoplasm, (2) pericytoplasmic and (3) perinuclear. The exposed embryos showed a pericytoplasmatic pattern in contrast to unexposed embryos that revealed a perinuclear and diffuse pattern (Figure 2). There was no significant difference between the ratios of Hoechst 33342-stained nuclei and total cells in good-quality among exposed and unexposed groups (Table 3; Figure 2).

**Table 2:** Comparison of fluorescence intensity among the unexposed and exposed bovine embryos to BoHV-5 suspension after MitoTracker Green FM and Jc-1 staining

**Groups**	**Total number of embryos**	**Number of embryos intensively labeled ± sd**	
		**MitoTracker Green FM**	**Jc-1**
*Unexposed*	150	53 ± 3.9^a^	40 ± 4.5^a^
*Exposed*	150	123 ± 8.5^b^	143 ± 8.2^b^

^a,b^ Within a column, values without a common superscript differ (*p* < 0.05).

**Table 3:** Comparison of the number of nuclei in unexposed and exposed of bovine embryos after Hoechst 33342 staining

**Groups**	**Number of embryos**	**Number of embryos mean ± sd**
*Unexposed*	150	107 ± 10.2^a^
*Exposed*	150	85 ± 4.5^a^

^a,b^ Within a column, values without a common superscript differ (*p* < 0.05).

### Antigens and genes related to mitochondrial activity, antioxidant protection and the stress response

**Figure 3:**
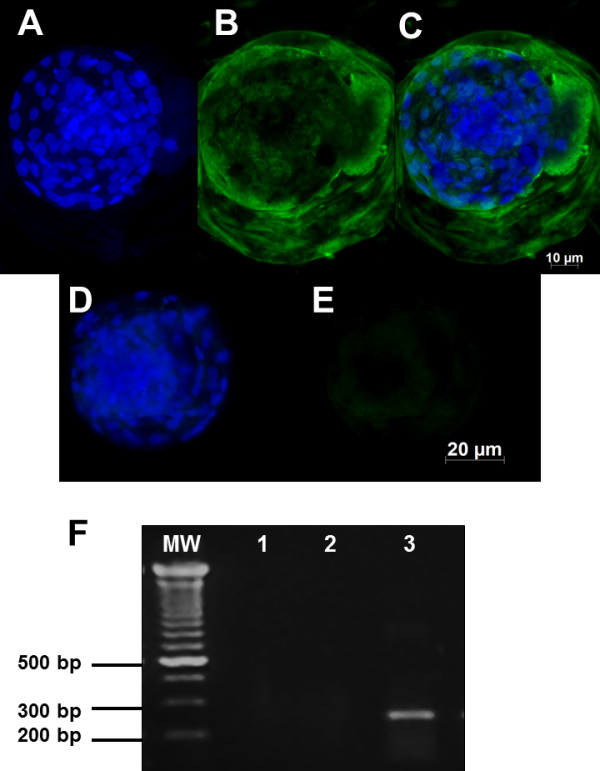
**A representative picture showing the total numbers of cells examined under fluorescence (FITC, 512 nm) for*superoxide dismutase 1 (SOD1).*** Day 7 bovine blastocysts derived from embryos exposed to BoHV-5 infection (**A-C**) and embryos that were unexposed (**D-E**) showing marked to mild intensity of SOD1-positive signals; **F**) electrophoresis agarose gel of SOD1 RT-PCR: MW-molecular weight 1-kb plus; lane 1 represents RT-PCR results from pure water used as a negative control; lane 2 shows the negative RT-PCR amplification of 7 d unexposed embryos; lane 3 shows the positive RT-PCR amplification of exposed embryos (297 bp). Scale bar is equal to 100 μm.

**Figure 4:**
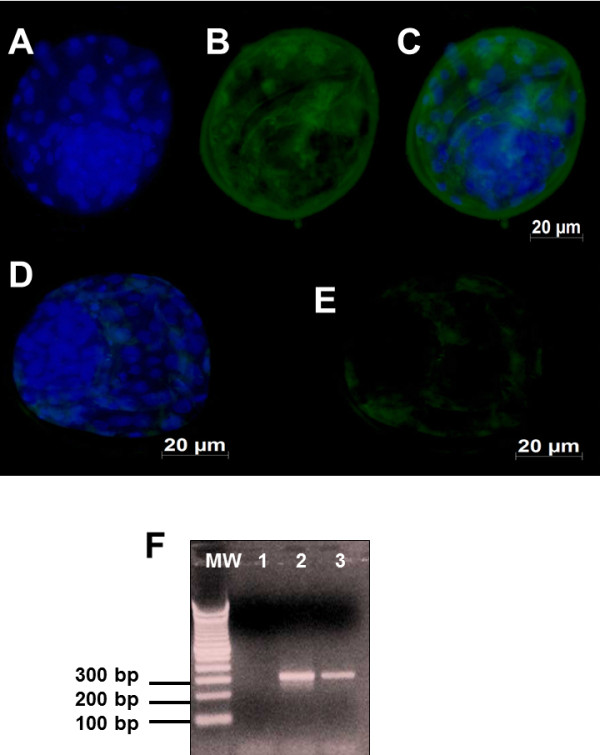
**A representative picture showing total numbers of cells examined under fluorescence (FITC, 512 nm) for AOP-1: A-C) Day 7 bovine blastocysts derived from oocytes exposed to BoHV-5 infection showing marked signals to AOP-1; D-E) Day 7 blastocysts derived from oocytes unexposed to BoHV-5 infection showing mild signals to AOP-1.** Electrophoresis agarose gel of AOP-1 RT-PCR: MW-molecular weight 1-kb plus; lane 1 represents RT-PCR results from pure water used as a negative control; lane 2 shows the AOP-1 negative RT-PCR amplification of unexposed embryos (310 bp); lane 3 shows the positive RT-PCR amplification of exposed embryos (310 bp). Scale bar is equal to 100 μm.

The relative expression of each cell marker and gene transcript studied, *SOD1, AOP-1* and *Hsp70* is depicted in Figures 3 and 4. Embryos exposed to BoHV-5 had an increased expression of *SOD1* (Figures 3A-C) compared with the unexposed group (Figures 3D and E). *SOD1* gene transcription was only detected in exposed embryos (Figure 3F; line 3). The *AOP1* antigens were detected in high amounts in the pericytoplasm and perinuclear regions of the exposed embryos (Figures 4 A-C). However, *AOP-1* transcription was observed in both groups of exposed and unexposed embryos (Figure 4F, lines 3 and 4). No detectable signal or positive amplicon for Hsp70 protein was obtained in this study.

## Discussion

The successful replication of a virus within a cell requires a remarkable cascade of interactions between virus and host. As part of their arsenal, many viruses maintain the ability to modulate cell metabolism to produce viral particles [7,10,23,28].

Experimental infection of bovine embryos with BoHV-5 in this study appeared not to interfere with the in vitro production of embryos. These results are consistent with our previous results that also showed that BoHV-5 exposure had no impact on the development in vitro *of* bovine embryos [11,12]. These observations contrast with the findings that BoHV-1 directly affects the fertilization process, supported by the observation of a strong decrease in the embryonic development rate when bovine IVF was performed in the presence of BoHV-1 [1]. Therefore, the potential of BoHV-5 to induce bovine reproductive disorders should not be underestimated, and a review of the sanitary measures required for international marketing of biological products should be considered [21].

In this respect, the regulation of the expression of genes related to the stress response, such as heat shock proteins (*Hsp*), could also be affected by different embryonic densities and inadequate culture conditions [4]. This condition was excluded in the present investigation because the exposed and unexposed embryos did not express mRNA and antigens related to *Hsp 70.1.* As the embryos produced for this analysis were not under stress caused by the IVC system, other parameters could be directly compared based on BoHV-5 exposure.

The abundance of *SOD1* antigens and the respective transcripts were not similar in both groups of embryos. Some studies revealed that higher levels of *SOD1* transcription may be an indicator of lower mitochondrial activity [5]. Furthermore, differences in oxygen concentration may also contribute to altered mitochondrial activity [6]. The IVC system applied in this study did not appear to affect the embryonic development. However, physiologically, the abundance of *SOD1* mRNA tended to decrease from the zygote to the initial blastocyst stage [8,9]. However, the correct sequence of this event is not fully understood. In addition, *SOD1* gene expression has been associated with good-quality, in vitro-produced embryos [9]. Moreover, an increase in *SOD1* expression has been related to a reduction in both the number of apoptotic cells and atresic follicles in mouse ovaries, showing that this gene is related to cell quality [3].

Recently, it has been described that bovine oocytes exposed to BoHV-5 after embryonic development show a reduction in the number of apoptotic cells [3]. Another parameter to be considered is the higher fluorescence intensity of MitoTracker Green FM probe, as well as Jc-1 labeling, among the exposed embryos. These two dyes are considered to be important for the qualitative and quantitative measure of mitochondrial activity [7]. In fact, mitochondria itself do not multiply until embryo hatching. These organelles are responsible for energy production until genome activation begins. According to a previous study, the blastocyst hatch is characterized by a mitochondrial distribution pattern changing from perinuclear to pericytoplasmatic localization, while in the cells of the inner mass, the distribution remains unchanged [9]. This pattern was observed in embryos exposed to BoHV-5, where the inner mass was intensively labeled by MitoTracker Green FM probe compared with unexposed embryos, and the label was more evident in the pericytoplasmatic area. Moreover, the exposed embryos were also intensively labeled by the Jc-1 probe, demonstrating pericytoplasmatic localization, which was in contrast with the unexposed embryos that were markedly labeled in the inner mass compartment. These patterns are considered physiological since trophoblastic cells start expressing adhesion proteins and other molecules important for maternal recognition of the embryos [9]. One possible explanation for the intense mitochondrial activity among exposed embryos is that virus replication demands more energy than usual. However, this activity does not seem to interfere with embryonic development. Taken together, these observations suggest that exposure of bovine embryos to BoHV-5 increases mitochondrial activity without any adverse physiological effect on the developing embryo, an important characteristic among the viruses from the *Herpesviridae*[10,28].

Reactive oxygen metabolites (ROS) appear to play a role in the cause and progression of several reproductive events both in humans and animals, such as fertilization and early development [5,7]. Recently, oxidative stress has been proposed as responsible for and as a possible cause of embryonic mortality in dairy cows [29]. The oxidative stress condition is thought to result from an imbalance between the production of ROS and the neutralizing capacity of antioxidant mechanisms [7]. However, the significant induction of ROS or the depletion of cellular antioxidants induces cell death, and ROS are likely to act as signaling intermediates that are involved in the signal transduction mechanism of apoptosis [3,22,23]. The (*AOP-1*) functions as a thioredoxin-dependent peroxidase that scavenges ROS, such as H_2_O_2_[30,31]. Remarkably, we were only able to detect the antigens and mRNA of *AOP-1* among embryos exposed to BoHV-5. The role of *AOP-1* in viral infections has previously been demonstrated; however, this is the first report of the association between *AOP-1* and in vitro-produced bovine embryos [30-32].

In summary, this study confirmed that BoHV-5 is capable to infect bovine oocytes. Furthermore, we conclude that experimental infection of oocytes with BoHV-5 does not interfere with cell viability, which facilitates viral replication by modulating mitochondrial function and producing antioxidative factors. However, multiple mechanisms are involved in cell survival during viral infection, and the majority of these mechanisms remain unclear.

## Conclusions

This is the first demonstration of *SOD1* and *AOP-1* production in bovine embryos exposed to BoHV-5. Intense mitochondrial activity was also observed during infection, and this occurred without interfering with the quality or number of produced embryos. These findings further our understanding on the ability of α-*Herpesviruses* to prevent apoptosis by modulating mitochondrial pathways.

## Competing interests

The authors declare that they have no competing interests.

## Authors’ contributions

MCPB and C SF prepared the in vitro-produced embryos and performed the exposure to BoHV-5. MCF performed the IFA and RT-PCR. AFG discussed the mitochondrial functions and statistical analyses. TCC and EFF were responsible for the funding and performed the final version of the manuscript. All authors read and approved the final manuscript.
